# CDC20 Knockdown and Acidic Microenvironment Collaboratively Promote Tumorigenesis through Inhibiting Autophagy and Apoptosis

**DOI:** 10.1016/j.omto.2020.03.015

**Published:** 2020-03-30

**Authors:** Qingying Gu, Fang Li, Shengfang Ge, Feifei Zhang, Renbing Jia, Xianqun Fan

**Affiliations:** 1 Department of Ophthalmology, Ninth People’s Hospital, Shanghai Jiao Tong University School of Medicine, Shanghai, China; 2 Shanghai Institutes for Biological Sciences, University of Chinese Academy of Sciences, Chinese Academy of Sciences, Shanghai, China; 3 Shanghai Key Laboratory of Orbital Diseases and Ocular Oncology, Shanghai, China

**Keywords:** CDC20, acidic microenvironment, tumorigenesis, metabolism, autophagy

## Abstract

The reconstitution of the tumorigenesis process would shed light on the tumor development study and further drug selection strategies. To construct a tumorigenesis model and explore potential mechanism is of great importance. In our study, we observed that CDC20-knockdown cells cultured in acidic environment exhibited chromosomal instability and better survival ability. The tumorigenic metabolism transformation was confirmed through the increase of the extracellular acidification rate (ECAR) and decrease of the oxygen consumption rate (OCR) in CDC20-knockdown cells. After a long-term culture for 3–4 months, CDC20-knockdown cells in acidic medium showed a strong tumor formation ability by subcutaneous injection into mice that is similar to that of tumor cells. Meanwhile, transcriptome analysis of cells from different stages showed that stage D cells almost resembled the phenotype of immortal cancer cells. The oncogene accumulation laid a firm foundation in the development of the tumorigenesis process by suppressing autophagy and p53-induced apoptosis. Several autophage- and apoptosis-related genes showed inhibition during this tumorigenesis process. In summary, chromosomal instability induced by CDC20 knockdown and acidic microenvironment could collaboratively promote cell tumorigenesis through the downregulation of autophagy and apoptosis.

## Graphical Abstract

**Figure:**
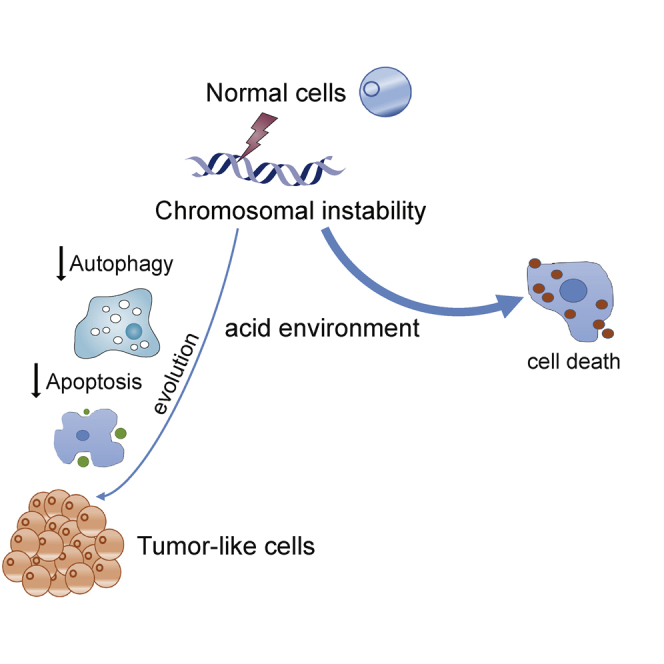

## Teaser

CDC20 knockdown could induce chromosomal instability. CDC20-knockdown cells cultured in acidic microenvironment could promote cell tumorigenesis. The tumorigenesis process was caused by downregulation of autophagy and apoptosis.

## Introduction

The tumor microenvironment is a complex physical and biochemical environment that characterizes solid cancers. It has attracted increasing attention after researchers identified potential relationships between oncogenic mutations and the role of these mutations as drivers of metabolic transformation.^1^ Highly proliferative cancer cells create tumor masses that lack nutrients and oxygen because of their increasing distance from blood vessels. The stabilization of the transcription factor hypoxia inducible factor 1 in response to hypoxic stress represents an important mechanism regulating the transition to glycolytic metabolism.^2^ In cancer cells, glucose is metabolized to pyruvate and lactate instead of entering the oxidative phosphorylation pathway, even under normal oxygen pressure, causing the Warburg effect.^3^

Enhanced glucose metabolism inevitably results in high yields of metabolic acids (lactate and protons) that cells must export to avoid intracellular acidification. The acidic microenvironment in tumor cells is an important component that drives tumor invasiveness, neovascularization, anchorage-independent growth, and genetic instability, which together contribute to malignant progression.^4^ Although metabolic reprogramming is clearly necessary to support tumor growth, the factors that initially induce a cell to rewire its metabolism have not yet been completely elucidated. Some clues have been provided by cells with an autophagy deficiency caused by abnormal mitochondria. Other pathways have been implicated in mediating the metabolic shift during cancer, including the tumor suppressor p53, which maintains the transcription of cytochrome *c* oxidase subunits and subsequent functional respiration by synthesizing cytochrome *c* oxidase 2.^5^ CDC20, whose activation promotes the activation of the anaphase-promoting complex/cyclosome (APC/C), is an important regulator of the duration of mitosis. The knockdown of CDC20 would cause chromosome segregation, which is a kind of chromosomal instability (CIN) commonly observed in solid tumors. To find out the collaborative effect of acid environment and CIN, CDC20 was knocked down in our study, and cells were cultured in a tumor-like microenvironment in an attempt to model the tumorigenesis process. Our model was highly functional, and we identified some important targets for oncotherapy during the early phase of tumorigenesis.

## Results

### Construction of Cells with Induced CIN

**Figure 1:**
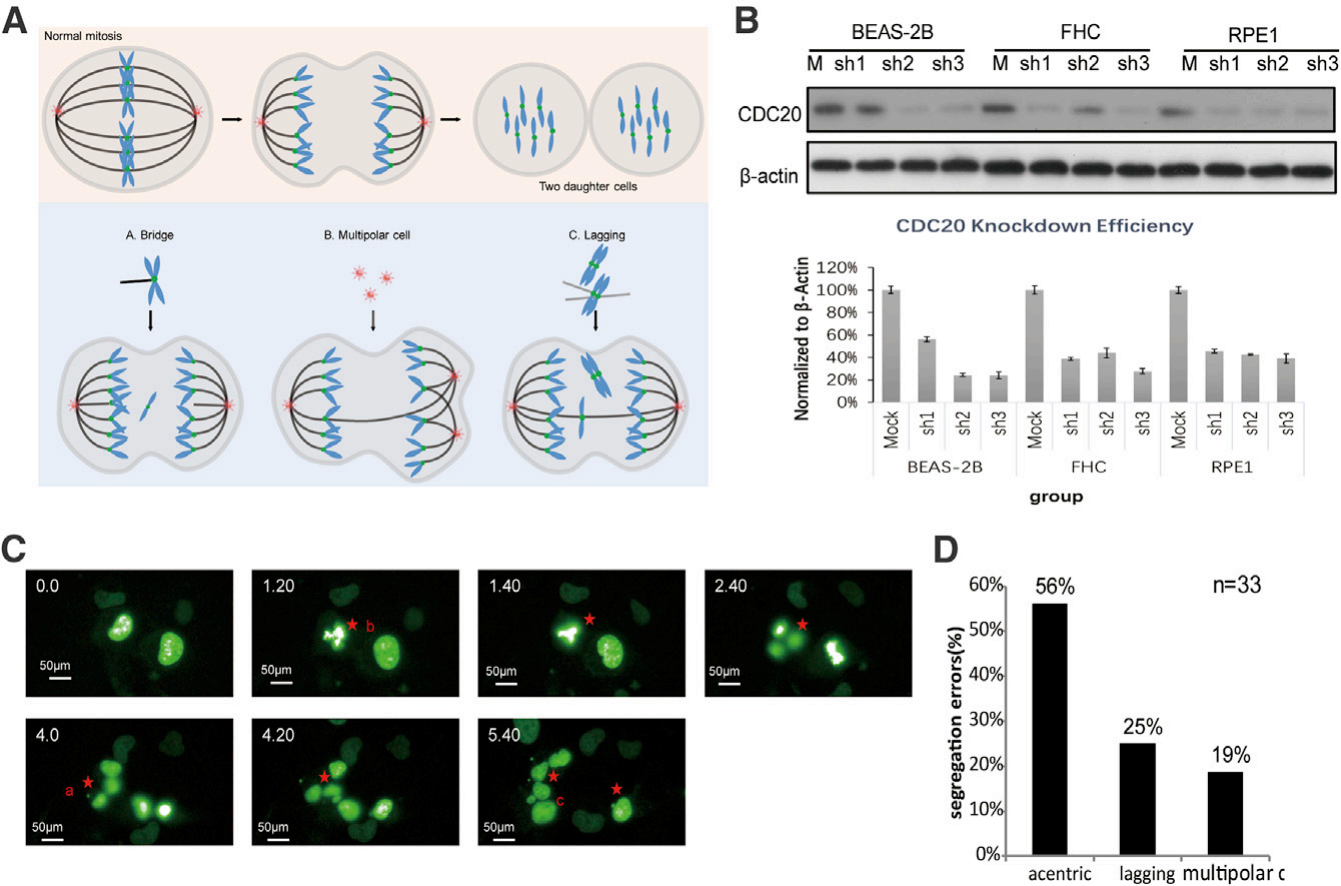
CIN Induced by CDC20 Knockdown in Normal Cells (A) Mitotic cells mis-segregate one or multiple chromosomes by generating mutations in the SAC pathway, premature loss of chromatid cohesion, transition via a multi-polar spindle, or merotelic attachment. (B) CDC20 silencing efficiency in three normal cell lines—BEAS-2B, FHC, and RPE1—using sh1, sh2, and sh3. The knockdown efficiency was statistically analyzed. All data are presented as mean ± standard error of the mean (SEM). (C) Images were captured from a live-cell experiment showing the mitosis process in RPE1 cells in which CDC20 expression was knocked down. (D) Percentage of segregation errors in micronuclei, multipolar cells, or anaphase bridges of CDC20^−^ RPE1 cells (n errors = 33; total n = 150). All subsequent experiments performed using cell lines were normalized to M and shC.

CIN refers to the alterations in chromosome number and structure that result in genomic instability, a hallmark of solid tumors. Due to the development of imaging technology, researchers have identified various mechanisms that result in genomic instability in the cell. During normal mitosis, chromosomes and the spindle replicate during interphase, the spindle fibers from opposite poles are attached to each sister chromatid on the same chromosome, all the chromosomes are arranged on the equatorial plate in neat rows during metaphase, the spindle assembly checkpoint (SAC) monitors whether the spindle fibers are correctly connected to the right centromere, and then each sister chromatid is properly translocated to the correct daughter cell during anaphase. Therefore, the destruction of checkpoints produces spontaneous mutations in cells that will have a high probability of being preserved and transferred to daughter cells. Thus, mitotic cells may mis-segregate one or multiple chromosomes by generating mutations in the SAC pathway, premature loss of chromatid cohesion, transitions via a multi-polar spindle, or merotelic attachment (Figure 1A).

We designed three lentiviral vectors expressing short hairpin RNAs (shRNAs), pLVX-Tight-puromycin-shCDC20, to construct CDC20-silenced cells and test our hypothesis. After incubation with 1 g/mL puromycin for two generations, the cells were collected for further verification. First, we performed western blots to verify the knockdown efficiency (Figure 1B); cells transfected with the empty vector were defined M, while CDC20-knockdown cells were defined shC, and all subsequent experiments used the most effective shRNA, shRNA-3 (Figures S1A and S1B). Second, we monitored the efficient progression of mitosis in knockdown cells. Knockdown cells transfected with pCMV-Tag1-H2B-EGFP were generated in advance to visualize the mitosis process. Then, the cells with green fluorescent chromosomes were subjected to time-lapse imaging using the PerkinElmer Operetta High Content System. Images were captured continuously to intuitively observe chromosomes during mitosis. Upon silencing CDC20 expression, increased CIN was monitored for 72 h with a camera (Figure 1C). Among all organisms analyzed to date, cells with impaired SAC function or defective cohesion still produce daughter cells (CIN) with micronuclei, because chromosome segregation occurs even when chromosomes are unattached or incorrectly attached (56% acentric). In addition, some cells also showed more than two centrosomes or fractured centrosomes (multipolar cells, 19%). Moreover, unequal merotelic attachments, resulting from kinetochores attached to more microtubules emanating from one pole than the other, are thought to missegregate, causing CIN (25% lagging; Figure 1D).

### CDC20-Silenced Cells Showed Increased Cell Growth after Short-Term Culture in the Acidic Environment

Accordingly, a significant chromosomal disorder was observed in CDC20-silenced cells that was very similar to early cancer phenotypes. Chromosomal disorders produce a substantial amount of unnecessary stress on cellular metabolism, such as oxidative stress and metabolic pressure; however, these seemingly meaningless mutations enable cells to survive in the tumorigenic microenvironment. We cultured cells displaying CIN in an acidic microenvironment, a typical characteristic of the tumor microenvironment, to simulate the process of tumorigenesis *in vitro* and confirm our hypothesis.

Under normal circumstances, normal cells do not grow in acidic environment. As the acidity increases, the growth of cells is significantly inhibited. If normal cells are continuously cultured in an acidic environment, they will eventually die. Surprisingly, cells with induced CIN survived for longer periods in an acidic environment than control cells, although these altered cells were also affected. In subsequent experiments, we performed a gradient acidity experiment to screen for the most suitable condition for long-term culture. Acidic media at pH 7.0, pH 6.6, and pH 6.2 not only increased the difference in proliferation between chromosomal unstable and control cells but also prevented excess pressure on cells (Figures S1C and S1D).

**Figure 2:**
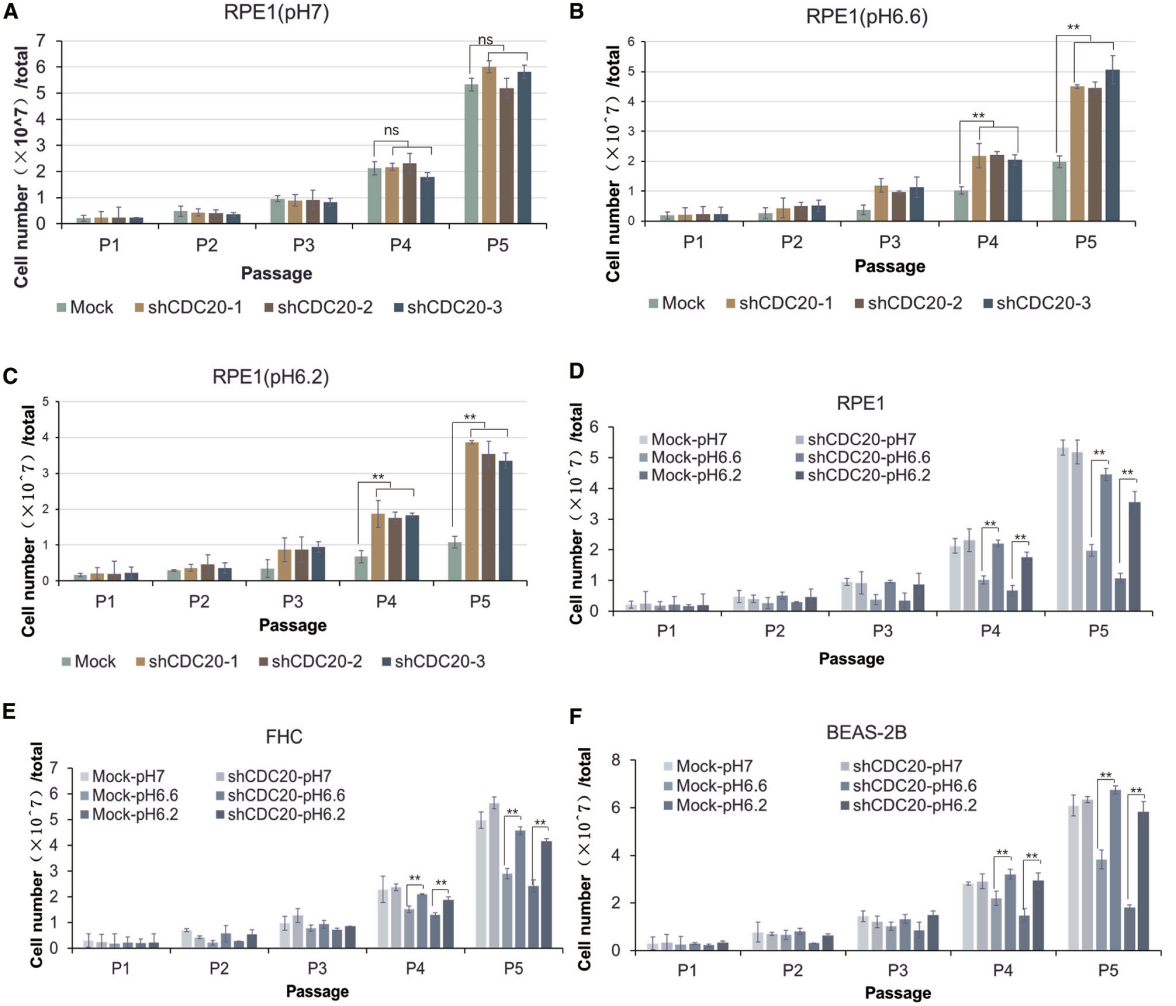
Growth Advantage of CDC20-Knockdown Cells in Acidic Environment Three normal cell lines (RPE1, BEAS-2B, and FHC) and their CDC20-knockdown variants were cultured in media at different pHs for 5 passages. Cell numbers were counted at each passage. (A) Numbers of RPE1 cells cultured in pH 7 medium for 5 passages. (B) Numbers of RPE1 cells cultured in pH 6.6 medium for 5 passages. (C) Numbers of RPE1 cells cultured in pH 6.2 medium for 5 passages. (D) Numbers of mock and shCDC20 RPE1 cells cultured in media at different pHs were counted and analyzed. (E) Numbers of mock and shCDC20 FHC cells cultured in media at different pHs were counted and analyzed. (F) Numbers of mock and shCDC20 BEAS-2B cells cultured in media at different pHs were counted and analyzed. All data are reported as the means ± standard error of the mean (SEM) of three biological replicates. ∗∗p < 0.01. ns, not significant.

As indicated in Figures 2A–2C, CDC20-knockdown cells exhibited increased proliferation compared with mock cells under different pH conditions. Additionally, the proliferation of mock RPE1 cells cultured at pH 6.6 was reduced 50%–70% compared with cells cultured at pH 7; cells cultured at pH 6.2 continued to proliferate slowly. However, upon the silencing of CDC20 gene expression, the growth inhibition phenomena were substantially ameliorated. Acute exposure to pH 6.2 medium provoked much less inhibition of the proliferation of CDC20-knockdown cells. The three normal cell lines were subjected to the same conditions in the low-pH medium. Overall, the cell proliferation decreased as the pH decreased, and the three CDC20-knockdown cell lines all showed a significant growth advantage in response to different pH conditions (Figures 2D–2F). Thus, these cell lines represent an appropriate model for studying the mechanisms by which normal cells adapt, survive, and even grow in acidic conditions.

### CDC20-Silenced Cells Showed Metabolic Reprogramming after Short-Term Culture in the Acidic Environment

Another important feature of tumorigenesis is metabolic reprogramming, known as the Warburg effect. Tumor cells typically divert pyruvate and its precursors to fuel other anabolic processes or convert these molecules into lactate for excretion from the cell. This metabolic adaptation is known as the Warburg effect. As described earlier, CIN cells adapt to acidic microenvironments more quickly and efficiently and, thus, exhibit better survival than normal cells cultured in acidic media with various levels of DNA damage. As the acidic microenvironment is identical to the tumor microenvironment, we postulated that CIN cells passaged in acidic medium 5 times may adapt to the condition and become early tumor-like cells.

We examined changes in aerobic glycolysis in CDC20-silenced cells and control cells using Seahorse XF analyzers to evaluate whether CIN cells altered their metabolism in the presence of acidic medium to resemble early tumor-like cells. The oxygen consumption rate (OCR) reflects mitochondrial glucose oxidation, and the decrease in oxygen respiration corresponds to an increase in glycolytic metabolism. The extracellular acidification rate (ECAR) represents the proportion of anaerobic respiration.

**Figure 3:**
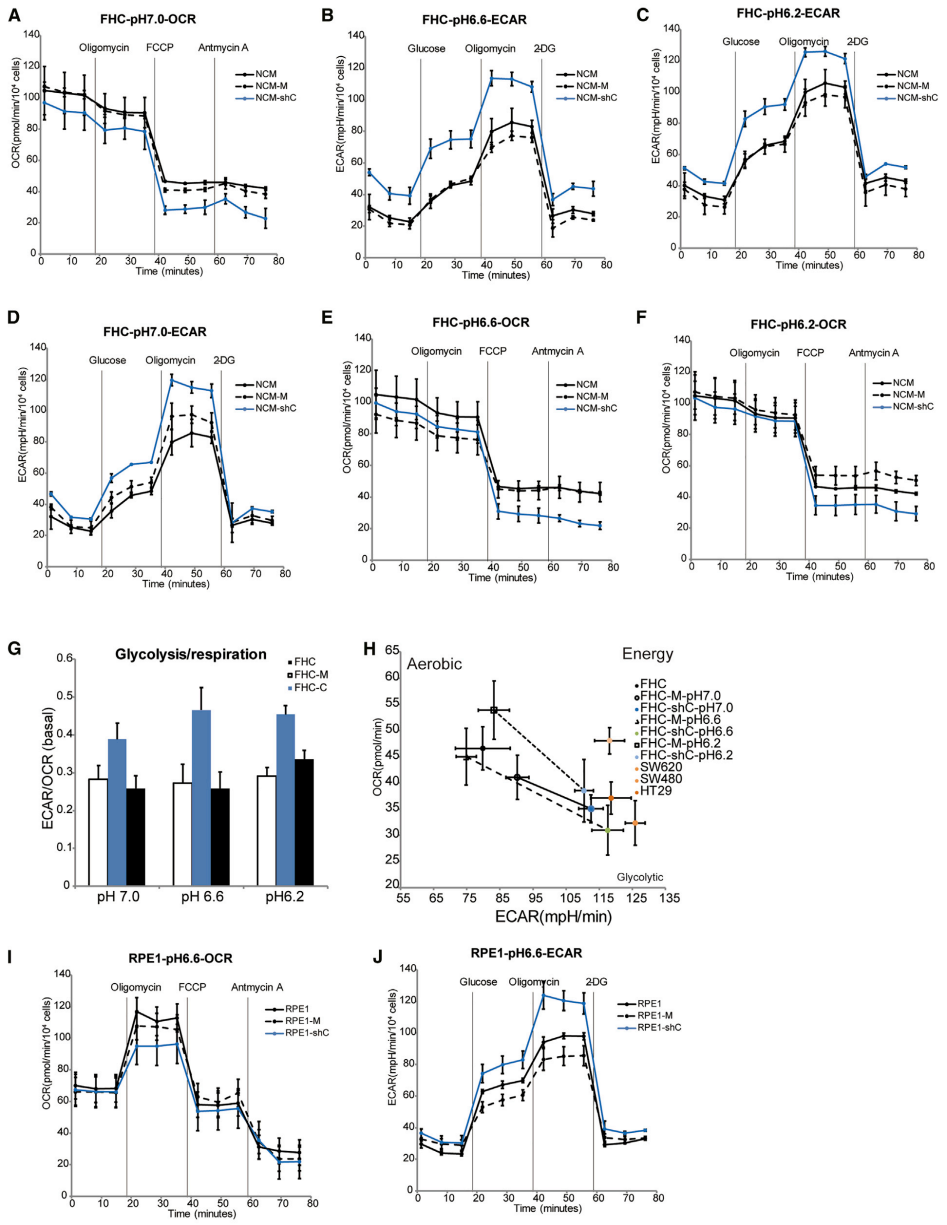
Metabolic Reprogramming of CDC20-Knockdown Cells Cultured in Acidic Environment Analysis of the ECAR and OCR in control, mock, and shCDC20 FHC cells cultured at different pHs. The data are presented as the means (±SEM) of triplicates. 2DG, 2-deoxy-D-glucose. (A) The glycolytic capacity of control, mock, and shCDC20 FHC cells cultured in pH 7 medium. (B) The glycolytic capacity of control, mock, and shCDC20 FHC cells cultured in pH 6.6 medium. (C) The glycolytic capacity of control, mock, and shCDC20 FHC cells cultured in pH 6.2 medium. The mitochondrial respiration of control, mock, and shCDC20 FHC cells was measured by determining the OCR. (D) The mitochondrial respiration of control, mock, and shCDC20 FHC cells cultured in pH 7 medium. (E) The mitochondrial respiration of control, mock, and shCDC20 FHC cells cultured in pH 6.6 medium. (F) The mitochondrial respiration of control, mock, and shCDC20 FHC cells cultured in pH 6.2 medium. (G) The glycolytic capacity and mitochondrial respiration of control, mock, and shCDC20 FHC cells cultured in pH 7.0, pH 6.6, and pH 6.2 media were analyzed, and the ratios are shown. (H) The total metabolic phenotype was measured by determining the ECAR and OCR in the same cells using the metabolic phenotype map. FHC cells cultured in different acidic environments and typical colorectal cancer cells were analyzed. The ratio of glycolysis and respiration was analyzed. These results are representative of three independent experiments. (I) The glycolytic capacity of control, mock, and shCDC20 RPE1 cells cultured in pH 6.6 medium. (J) The mitochondrial respiration of control, mock, and shCDC20 RPE1 cells cultured in pH 6.6 medium.

As shown in Figure 3A, the OCR of CDC20-knockdown cells was significantly decreased compared with that of mock cells and control cells in pH 7 medium. Consistent with the result obtained at pH 7.0, the OCR also decreased in pH 6.6 medium and pH 6.2 medium (Figures 3B and 3C). Among these changes, the OCR exhibited the most substantial decrease in pH 6.6 medium. CDC20-knockdown cells displayed a significant increase in the ECAR compared with mock cells and control cells cultured in pH 7 medium (Figure 3D), indicating that CDC20 silencing decreased oxygen respiration and increased glycolytic metabolism compared to normal cells. Consistent with the ECAR of FHC cells cultured in pH 7.0 medium, cells in cultured pH 6.6 and 6.2 media also showed the same trends (Figures 3E and 3F). In addition, the ECAR and OCR were also simultaneously measured using the Seahorse XF Cell Energy Phenotype Test Kit to test the two major energy-producing pathways, mitochondrial respiration and glycolysis, in live cells. This result showed an increase in the ECAR/OCR ratio in CDC20-knockdown cells compared with mock cells (Figure 3G). Regarding the different pH conditions, we also observed an increased ECAR and decreased OCR in CDC20-knockdown FHC cells in a pH-dependent manner; similar findings were obtained from SW620, SW480, and HT29 cells. This result also indicated a metabolic switch from normal cells to tumor-like cells (Figure 3H). In addition to FHC cells, we also performed the test in RPE1 cells, and the same trends were observed in RPE1 cells cultured in pH 6.2 medium (Figures 3I and 3J).

### CDC20-Silenced Cells Formed Tumors after Long-Term Culture in the Acidic Environment

**Figure 4:**
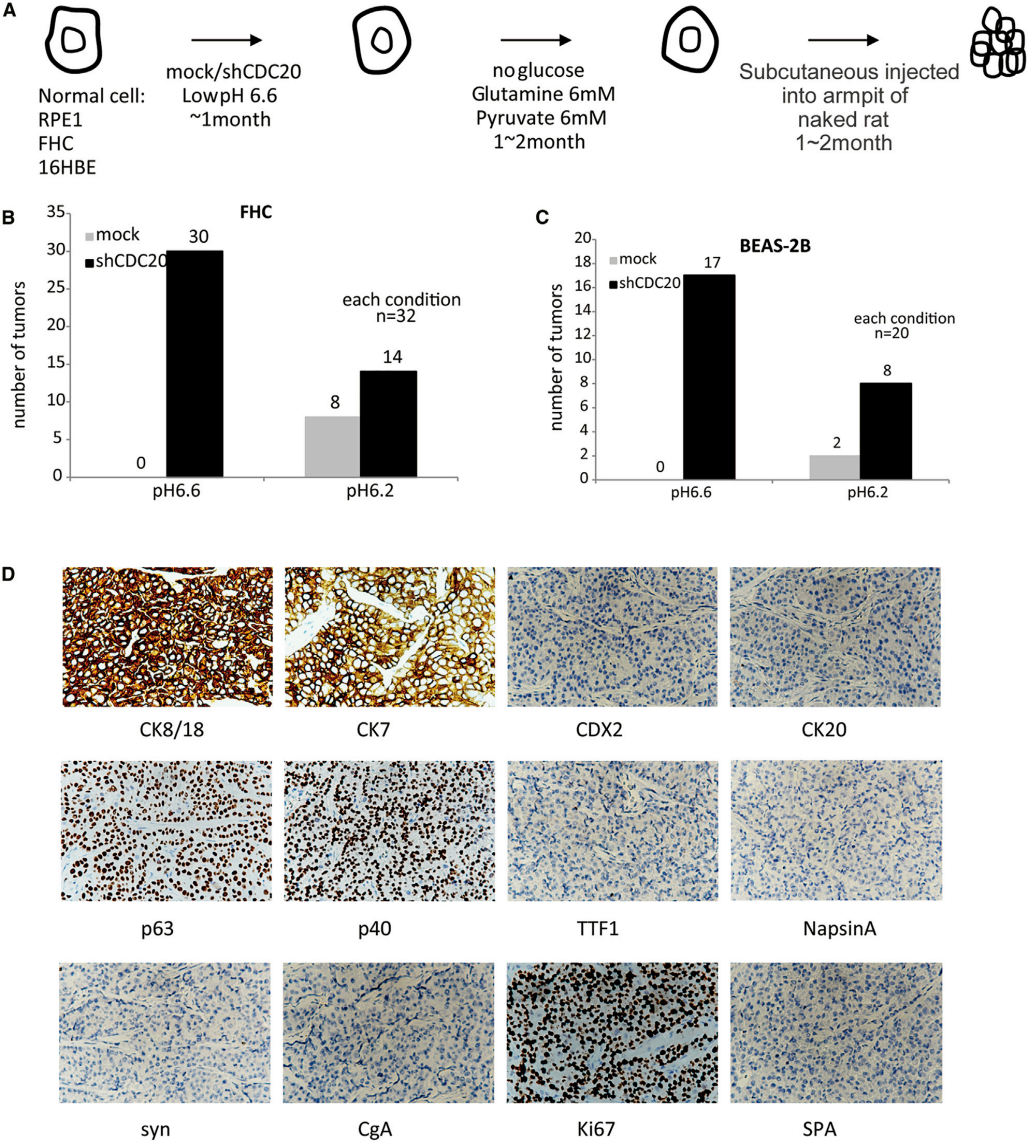
Tumor Formation of CDC20-Knockdown Cells after Long-Term Culture in Acidic Environment (A) The procedure used for the long-term culture of CDC20-knockdown cells in an acidic environment. First, CDC20 stably knockdown cells were prepared and cultured in acidic medium for 1 month. Then, cells were cultured in medium containing high concentrations of glutamine and pyruvate for 1–2 months. Cells showing significantly increased growth were injected into the mice to determine the tumor formation capability. Meanwhile, cells were collected at different stages, and RNA-seq was performed to identify the changes in transcriptome. The tumors that formed after stage-D cells were injected into nude mice. (B) The number of tumors formed from FHC cells cultured in media at different pHs was statistically analyzed. (C) The number of tumors formed from BEAS-2B cells cultured in media at different pHs was statistically analyzed. (D) Immunohistochemical staining for classical tumor markers in tumors formed from cells cultured in pH 6.6 medium. The markers included CK8/18, CK20, CDX2, CK20, p63, p40, TTF1, Napsin A, Syn, Cg-A, Ki67, and SPA.

We optimized the conditions of the tumor microenvironment and constructed a low-nutrient environment to mimic short-term stress and further determine whether the culture process promoted tumorigenesis. The process used for the long-term gradual culture of chromosomal unstable cells is shown in Figure 4A. First, stable CDC20-knockdown cells were prepared and cultured in acidic medium for 1 month. Then, the cells were cultured in medium containing high concentrations of glutamine and pyruvate for 1–2 months. Cells showing significantly increased growth were injected into mice to test their tumorigenic activity. Meanwhile, cells were collected at different stages, and RNA sequencing (RNA-seq) was performed to identify changes in the transcriptome. CDC20-knockdown cells subjected to long-term culture in acidic medium formed obvious tumors. The statistical analysis of the results is shown in Figure 4B. CDC20-knockdown FHC cells cultured in pH 6.6 medium formed many more tumors than mock cells cultured in the same medium. Moreover, CDC20-knockdown cells cultured in pH 6.2 medium formed more tumors than normal cells but formed fewer tumors than cells cultured in pH 6.6 medium, consistent with the results from our cell-growth experiment described earlier. The knockdown of CDC20 in BEAS-2B cells also produced the same result. CDC20-knockdown BEAS-2B cells formed many more tumors than mock cells cultured in both pH 6.6 and pH 6.2 media (Figure 4C). Immunohistochemical staining confirmed that the tumors showed classical features of colorectal cancer. Several markers were identified, as shown in Figure 4D. The tumor was CK8/18(+), CK7(+), CDX2(−), CK20(−), p63(+), p40(+), TTF1(−), Napsin A(−), Syn(−), Cg-A(−), Ki67(+), and SPA(−), confirming that the tumor was a poorly differentiated squamous carcinoma.

### CDC20-Silenced Cells Showed Alterations in the Transcriptome after Long-Term Cultivation in the Acidic Environment

**Figure 5:**
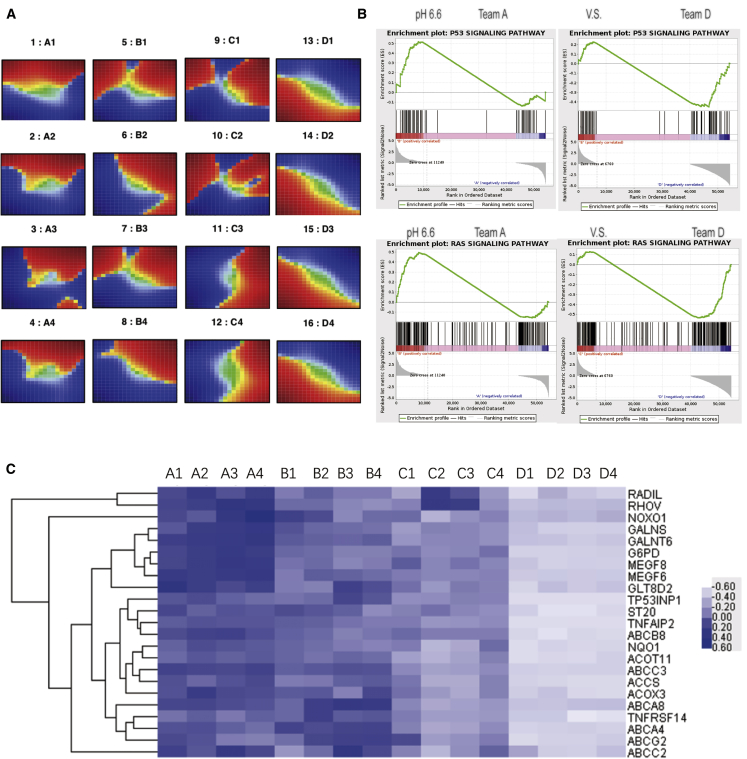
Transcriptome Analysis of CDC20-Knockdown Cells in Acidic Environment (A) Cells were collected at different stages, and transcriptome sequencing was performed. The SOM function was analyzed in cells collected at different stages. (B) GSEA of the p53 signaling pathway and RAS signaling pathway in stage-A and stage-D cells. (C) Heatmap of some differentially expressed genes.

Cells were collected at different stages, and transcriptome sequencing was performed. Four replicates collected at different stages all showed the same trends, as shown in Figure 5A. Additionally, the self-organizing map (SOM) function analysis showed important changes in the transcriptome from the first stage, A, to the fourth stage, D, and some important genes located throughout the transcriptome showed opposite changes. We also performed an in-depth analysis of the transcriptome sequencing data to validate the effectiveness of this model cultured in an acidic environment. Based on the results from the gene set enrichment analysis (GSEA), the p53 signaling pathway and RAS signaling pathway were both expressed at low levels during culture (Figure 5B). As shown in the heatmap, some important genes showed significant changes during culture (Figure 5C). For example, some cancer suppressor genes—such as the ABCG2 gene, ABCA4 gene, and MEGF6/8 gene—were expressed at significantly lower levels from stage A to stage D.

### CDC20-Induced CIN Cells Survive the Acidic Environment by Inhibiting Autophagy and Apoptosis

**Figure 6:**
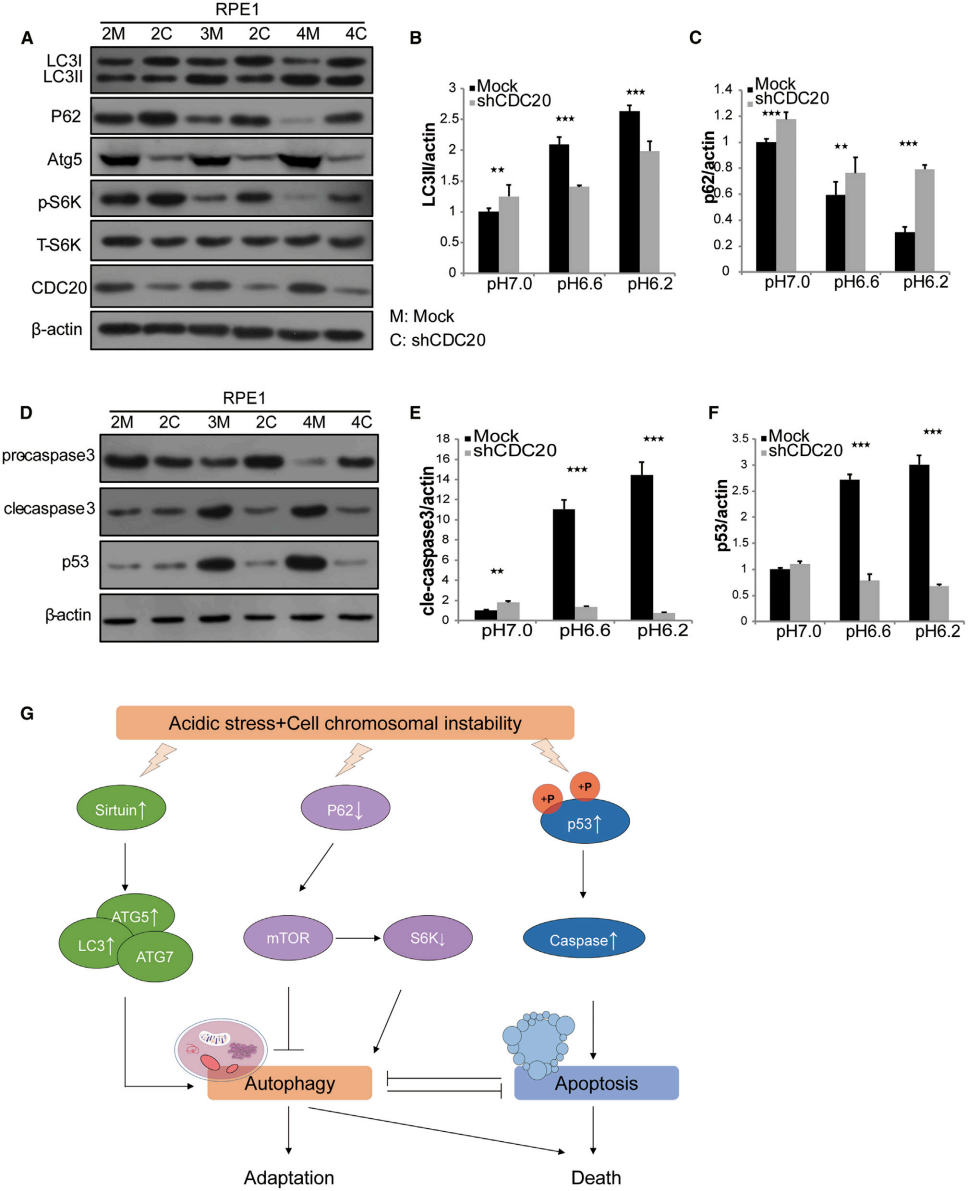
CDC20 Silencing in Acidic Environment Suppressed Autophagy and Apoptosis Mock RPE1 cells and shCDC20 RPE1 cells were cultured in various acidic media for 5 passages. Cells cultured in pH 7.0 media were normalized to 2M and 2C. Cells cultured in pH 6.6 media were normalized to 3M and 3C. Cells cultured in pH 6.2 media were normalized to 4M and 4C. (A) Levels of LC3, p62, ATG5, p-S6K, and S6K in mock and shCDC20 RPE1 cells cultured in different media were detected by western blotting. (B and C) The levels of (B) LC3 and (C) p62 were analyzed by western blotting and normalized to cells cultured in pH 6.7 medium (2 g/L MES). All data are presented as mean ± standard error of the mean (SEM). (D) Pro-caspase-3, cleaved caspase-3, and p53 levels in 2M, 2C, 3M, 3C, 4M, and 4C RPE1 cells were detected by western blotting. (E and F) Levels of (E) cleaved caspase-3 and (F) p53 were analyzed and normalized. All blots shown are representative of at least three independent experiments. All data are presented as mean ± standard error of the mean (SEM). (G) Potential mechanism by which cells survive acid and mitotic stress. In the presence of acid stress, autophagy and apoptosis may be induced to promote cell death. The survival of cells with CIN induced by CDC20 knockdown cultured in acidic media may be mediated by the inhibition of autophagy and apoptosis.

Because growth and metabolic reprogramming are short-term phenomena in response to stress, we tested the response of cells with a metabolic disorder. Autophagy is activated in metabolically stressed cells, and we speculated that this process might be a protective mechanism for cancer cells that are acutely exposed to acidic stress. We evaluated the effects of short-term exposure to acidic pH on basal autophagy in normal and knockdown cells to test this hypothesis. Interestingly, LC3-II levels were significantly increased in mock RPE1 cells as the pH of the medium decreased, while p62 and p-S6K levels were significantly decreased in mock cells after 5 passages in acidic medium in a pH-dependent manner. ATG5 expression was decreased and p-S6K levels were increased after CDC20 knockdown (Figure 6A). Quantification of protein expression revealed that mock RPE1 cells cultured in acidic pH medium exhibited increased LC3-II levels and decreased p62 levels, indicating that functional autophagy occurred under these conditions (Figures 6B and 6C). The same trend was also observed in FHC and BEAS-2B cells (Figures S1E and S1F). In addition to autophagy, apoptosis and senescence are other mechanisms by which cells may respond to acute changes in the microenvironment. Multiple different stressors activate p53 in the context of tumor initiation or progression. We also detected the levels of p53, pro-caspase-3, and cleaved caspase-3. The cleaved caspase-3 and p53 levels were increased in mock RPE1 cells in a pH-dependent manner. The cleaved caspase-3 and p53 levels were also decreased in CDC20-knockdown cells cultured in acidic media (Figure 6D). Quantification of protein expression revealed increased levels of cleaved caspase-3 and p53 in mock RPE1 cells cultured in medium with an acidic pH medium, indicating that functional apoptosis occurred under these conditions (Figures 6E and 6F). However, CDC20-knockdown cells did not undergo apoptosis, indicating a better survival rate. The same findings were obtained from FHC and BEAS-2B cells (Figures S1G and S1H).

According to our results, autophagy and apoptosis both played important roles in the culture process. In response to acid stress, the increase in ATG5 levels and decrease in p62 levels induced autophagy, thus inhibiting cell growth. However, the cell CIN induced by CDC20 knockdown combined with acid stress induced the abnormal accumulation of the autophagy substrate p62 in autophagy-deficient cells, and the abnormal S6K hyperphosphorylation subsequently suppressed autophagy, promoting the malignant transformation of CDC20-knockdown cells to tumor-like cells. Meanwhile, the increase in p53 levels promoted apoptosis, resulting in the death of cells cultured under acidic conditions. However, in the presence of CDC20-induced cell CIN and acid stress, apoptosis did not occur, thus revealing the mechanism underlying the increased growth of CDC20-knockdown cells (Figure 6G).

## Discussion

Here, CIN cells exposed to acute acid stress relied on various gene mutations and an acidic microenvironment to adapt and proliferate to a greater extent than normal cells. We provide evidence that acid stress induced by the acidification of the pH of the medium increased autophagic flux and apoptosis. The induction of various kinds of DNA damage by CDC20 silencing dramatically increased the resistance of normal cell lines to acid-stress-induced cell death, transforming normal cells to malignant tumor-like cells.

Let us first recall how chromosomes are segregated. When duplicated DNA strands (called sister chromatids) are linked by ring-like cohesin molecules, the cells begin to prepare for S-phase chromosome segregation.^6^ During prophase, sister chromatid pairs are attached to the mitotic spindle so that each sister kinetochore attaches to microtubules emanating from opposite spindle poles. In metaphase, sister chromatids are bi-oriented, and sister kinetochores form under tension by resisting microtubule and cohesin pulling forces. The SAC pathway monitors kinetochore attachment and tension and stops cell-cycle progression until every sister chromatid pair is properly attached to the mitotic spindle.^7^ SAC targets include the ubiquitin ligase APC/C, which binds to the APC-activating subunit CDC20 (APC/C^CDC20^). SAC inhibition is relieved when all sister chromatids are properly attached and the inhibitory subunit (securin) of the protease separase is degraded by APC/C^CDC20^. Then, cohesins are cleaved by active separase, initiating chromosome segregation.

We analyzed high-content images of the mitotic process in treated RPE1 cells to determine whether mitotic or pre-mitotic mechanisms are responsible for these segregation errors. Chromosome segregation occurred following CDC20 knockdown; and structural and numerical CIN, which are commonly observed together in solid tumors,^8^ were induced in the lab, as evidenced by pre-mitotic defects or by defective chromosome attachment to the mitotic spindle or mitotic checkpoint function that affects chromosome structure, resulting in faulty DNA repair and replication.^9^ CDC20 is the mitotic checkpoint and a cellular safeguard that prevents chromosome mis-segregation in eukaryotic cells,^10^^,^^11^ and suboptimal functioning of this checkpoint may promote chromosome mis-segregation in cancer cells.^12^ Checkpoint signaling produces the “mitotic checkpoint complex” (MCC), which prevents anaphase by targeting CDC20, the APC/C activator. In our study, CIN was also caused by CDC20 knockdown.

Environments with an acidic pH are known to delay growth and increase spontaneous apoptosis in cell lines derived from normal cells, such as lung and colon cells.^13^ However, tumor cells survive in the presence of acid stress. In our study, we observed decreased cell growth in response to acidic conditions, which was alleviated by CDC20 knockdown. The potential mechanism may be CDC20-knockdown-mediated inhibition of the autophagy and apoptosis of normal cells.

Under stressful conditions or conditions in which mutant proteins are expressed with increased levels of misfolded or aggregated proteins, autophagy plays an important role in eliminating protein aggregates.^14^ Autophagy limits genome instability, tissue damage, and inflammation, which promote cancer initiation, indicating that strategies that stimulate autophagy may be beneficial treatments to prevent cancer.^15^^,^^16^ Notably, p62 is a cytoplasmic protein that transports ubiquitinated proteins to autophagosomes. When autophagy is inhibited, the expression of p62 is increased, and the levels of p62 therefore reflect autophagic flux. The levels of ATG proteins or other proteins required for autophagy induction, such as sirtuin-1, are increased in autophagic cells; for example, ATG5 is upregulated in normal autophagosomes. Autophagy also results in AMPK activation and mTORC1 inhibition. The effects of mTOR inhibition on translation are mediated by the hypophosphorylation of its substrate, S6K. In our study, p62 and p-S6K levels were decreased in a pH-dependent manner after 5 passages in acidic medium, indicating that acid-dependent death may be mediated by autophagy. However, these trends were reversed in CIN cells (CDC20 knockdown), indicating that defective autophagy may be the mechanism underlying increased cell survival under acidic conditions.

Stalled or collapsed DNA replication forks recruit ataxia telangiectasia and RAD3-related (ATR), which phosphorylates CHK1, and a DNA double-stranded break triggers the activation of ataxia-telangiectasia mutated (ATM), a kinase that phosphorylates CHK2 kinase.^17^ The p53 protein is a substrate for ATM and ATR and matrix for both CHK1 and CHK2. In this situation, CHK1 and CHK2 comparably phosphorylate p53 (usually at serines 15 and 20) to promote its stabilization.^18^ The phosphorylation of p53 enhances its interaction with transcriptional cofactors, which will ultimately activate target genes and responses, such as DNA repair, cell-cycle arrest, apoptosis (caspase-3), and senescence.^19^

ATR and ATM also inhibit the p53 negative regulators MDM2 and MDM4. MDM2 serves as an E3 ubiquitin ligase to degrade p53, and MDM2 and MDM4 together bind to the transcriptional activation domains of p53 to inhibit its transactivation function.^20^ Furthermore, consistent with the predictions, a significant increase in the apoptosis of normal cells was observed in an acid-dependent manner. However, CDC20-knockdown cells showed reduced apoptosis levels, implying a much better survival and the malignant transformation of normal cells.

Metabolic reprogramming is considered a hallmark of cancer.^21^ Tumor cells tend to “ferment” glucose into lactate, even in the presence of sufficient oxygen to support mitochondrial oxidative phosphorylation, which is also known as the Warburg effect. Consistent with these findings, CDC20-knockdown cells cultured in acidic medium also showed a substantial decrease in the ECAR, a proxy for lactate production, and suppression of mitochondrial respiration (OCR) in the present study. These CIN cells gradually transformed into tumor-like cells after 5 passages in acidic medium.

The tumor microenvironment, gene mutations, and their subsequent stressors are major factors that contribute to cancer. While hypoxia and acidosis have long been described as two ubiquitous features of the tumor microenvironment, hypoxia has been studied more extensively.^22^ The concept of hypoxia-induced angiogenesis and the associated promise of new therapeutic methods^23^ and gene reprogramming driven by the well-characterized hypoxia-inducible factor (HIF)^24^ have greatly contributed to our knowledge of the function of tumor hypoxia in transforming genomes. Multiple studies reporting a correlation between an acidic tumor microenvironment and tumorigenesis, particularly local tumor invasion and distant metastatic spread, were published in the 1990s and early 2000s;^25^ insightful mathematical models of acid-mediated invasion were also reported.^26^ Moreover, the disruption of mitochondrial homeostasis is usually correlated with increased production of reactive oxygen species (ROS), powerful damaging agents that not only induce mutagenesis but also function as signaling molecules that contribute to cancer progression.^27^

Under these stresses, cells may utilize different mechanisms to promote survival. Cells sometimes promote cell-cycle arrest, DNA repair, apoptosis, and senescence responses to survive, while they may increase their autophagy levels to maintain homeostasis in other situations. Autophagy is the most important mechanism for the degradation and recovery of long-lived proteins, organelles, protein aggregates, and intracellular pathogens.^28^ Under normal physiological conditions, autophagy helps cells maintain homeostasis by “clearing” impaired/dispensable intracellular structures, thereby providing crucial protection against the accumulation of toxic cellular components. Consequently, autophagy serves as a protective response to various stress stimuli, suggesting a potential association between defective autophagy and several human pathologies, including cancer.^29^ When these recovery or suicide behaviors function normally, cells suppress tumor growth. However, when cells adapt to the stressful microenvironment and undergo metabolic reprogramming, their fate is likely very different. Metabolic reprogramming, a hallmark of cancer cells, is characterized by the Warburg effect, where high rates of glycolysis and reduced oxidative phosphorylation occur, even under aerobic conditions. This reprogramming is thought to be essential for fueling the anabolic processes required for the growth and proliferation of cancer cells.^30^ Thus, normal cells would adapt to the acidic microenvironment and become tumor-like cells.

In the present study, we cultured cells with induced CIN to allow them to adapt to the tumor microenvironment, thus constructing an *in vitro* tumorigenesis model. During culture with various pressure choices, the cells in the experimental group showed increased growth and proliferation, decreased expression of tumor suppressor genes, activation of oncogenes, and an accelerated speed of environmental self-adaptation due to dysfunctional autophagy. More importantly, in addition to the phenotype of an accelerated growth rate, the metabolism of cells was also reprogrammed, which is more similar to the Warburg effect of tumor cells. Finally, the normal cells were successfully transformed into tumor cells, as subcutaneous tumors formed in mice. Therefore, we postulate that the *in vitro* tumor induction model was effective and can be applied to drug screens.

In summary, the placement of normal cells with various random gene mutations in a tumor-like microenvironment may mimic the early tumorigenesis process *in vitro*. After 5 passages under multiple selective pressures, the normal cells analyzed here adapted to the microenvironment; they exhibited good growth, activated tumor suppressor genes, and exhibited impaired autophagy, among other phenotypes. More importantly, in addition to these proliferative phenotypes, the metabolic states of these cells tended to resemble those of tumor cells, which was a positive sign. Therefore, we validated this *in vitro* tumorigenesis model. Future studies will provide the additional evidence required to completely simulate the tumor microenvironment and facilitate a better understanding of the tumorigenesis process to therapeutically harness the power of this remarkable model.

## Materials and Methods

### Cell Lines and Cell Culture

The normal human cell lines FHC and BEAS-2B were purchased from the American Type Culture Collection (Manassas, VA, USA). The normal cell line RPE1 was obtained from the Cell Bank of the Chinese Academy of Sciences (Shanghai, China). All cell lines were cultured in RPMI-1640/DMEM/F12 medium supplemented with 10% fetal bovine serum (FBS; GIBCO, Life Technologies, Gaithersburg, MD, USA) and antibiotics (1 × 10^4^ U/L penicillin and 100 mg/L streptomycin) at 37°C in a humidified atmosphere of 95% air with 5% CO_2_, according to the supplier’s instructions. The low-pH medium (pH 5.5–6.5) was generated by adding 50 g/L MES (M2933, Sigma, Darmstadt, Germany), 2 mL MES (2 g/L; pH 6.7), 3 mL MES (3 g/L; pH 6.2), and 4 mL MES (4 g/L; pH 5.8) to 50 mL medium. To establish chronic low-pH exposure, we continuously cultured the cells at pH 5.5–6.5 for 5 passages (3 days for one passage) and counted the number of cells after each passage to establish chronic low-pH exposure conditions. Cells were maintained in low-pH and control-pH media in all experiments.

### Mammalian Lentiviral shRNAs

Lentiviral shRNA expression vectors were designed and purchased from Sigma-Aldrich. These shRNAs were inserted into the lentiviral packaging plasmid pLVX-Tight-Puro (a lentiviral vector used to express a gene with the Tet-On Advanced or Tet-Off Advanced system) to knock down target genes in the presence of 1 ng/μL doxycycline. Lentiviruses carrying the genes of interest were generated by cotransfecting HEK293T cells with envelope (vesicular stomatitis virus glycoprotein [VSV-G]) and packaging (Delta 8.9) plasmids using Lipofectamine 2000 (Invitrogen), according to the manufacturer’s instructions. The viral supernatants were harvested and filtered on day 2 post-transfection. Three normal cell lines were infected with the lentivirus in the presence of serum-containing medium supplemented with 8 μg/mL polybrene. Forty-eight hours after the infection, cells were selected with 2.0 μg/mL puromycin (Sigma). Knockdown efficiencies were confirmed by western blotting.

H2B-GFP was constructed by PCR from normal cell lines, and the product was cut with the selected restriction enzyme. The H2B-GFP cDNA was inserted into the retrovirus packaging plasmid pCMV-Tag1. A retrovirus carrying the H2B-GFP cDNA was generated by transfecting 293FT cells with the appropriate plasmid. RPE-1-knockdown cells were infected with the H2B-GFP virus for 48 h in the presence of 8 μg/mL polybrene, washed, and allowed to recover for 24 h before fluorescence-activated cell sorting.

### Western Blotting

Proteins were electrophoresed and transferred to polyvinylidene fluoride (PVDF) membranes. Membranes were incubated with a rabbit polyclonal anti-mouse/human CDC20 antibody (1:1,000; Proteintech, Rosemont, IL, USA), a rabbit polyclonal anti-mouse/human LC3 antibody (1:1,000; Novus, Shanghai, China), a mouse monoclonal anti-human p62 antibody (1:1,000; Abcam, Cambridge, MA, USA), a rabbit monoclonal anti-human ATG5 antibody (1:1,000; Abcam, Cambridge, MA, USA), a rabbit polyclonal anti-human p-S6K antibody (1:1,000; Cell Signaling Technology, Beverly, MA, USA), a rabbit polyclonal anti-human S6K antibody (1:1,000; Cell Signaling Technology, Beverly, MA, USA), a rabbit monoclonal anti-active caspase-3 antibody (1:1000; Abcam, Cambridge, MA, USA), a rabbit polyclonal anti-caspase-3 antibody (1:1,000; Abcam, Cambridge, MA, USA), or a mouse monoclonal anti-human β-actin antibody (1:1,000; Proteintech, Wuhan, China). Membranes were then subjected to immunodetection.

### Determination of the ECAR and OCR

The cellular glycolytic capacity and mitochondrial function were measured using the Seahorse XF Glycolysis Stress Test Kit and Seahorse XF Cell Mito Stress Test Kit on the Seahorse Bioscience XF96 Extracellular Flux Analyzer, according to the manufacturer’s instructions. Cells were seeded on an XF96 Cell Culture Microplate (Seahorse Bioscience) at a density of 2,000 cells per well and then incubated with acidic medium for 24 h. On the day before the assay, the cartridge sensor was hydrated with 1 mL Seahorse Bioscience XF96 Calibration Buffer overnight at 37°C without CO_2_. On the day of the assay, the growth medium was replaced with serum-free DMEM lacking sodium bicarbonate, and the cells were incubated at 37°C in a non-CO_2_ incubator for 1 h. The OCR and ECAR were monitored under basal conditions and measured after successive injections of the OCR compounds (1 μM oligomycin, 1 μM FCCP, and 0.5 μM antimycin A) or ECAR compounds (10 mM glucose, 1 μM oligomycin, and 50 mM 2-deoxy-D-glucose [2DG]) into the well. Data were normalized to the number of cells per well at the end of the culture period and expressed as the OCR in picomoles per minute and ECAR in milli-pH (mpH) units per minute, and the results were analyzed using the Seahorse XF96 software.

The simultaneous effects of ECAR and OCR on metabolic switching were measured using the Seahorse XF96 Cell Energy Phenotype Test Kit. Cells were seeded on an XF96 Cell Culture Microplate (Seahorse Bioscience) at a density of 2,000 cells per well, incubated overnight, and subjected to the procedures described earlier. The difference is that this test measures the utilization of each pathway, mitochondrial respiration, and glycolysis, first under the starting medium conditions (baseline phenotype) and then upon the injection of a stressor mix (1 μM oligomycin and 1 μM FCCP) that induces an energy demand (stressed phenotype). The Seahorse XF Cell Energy Phenotype Test Report Generator plots these data on the energy map and displays the metabolic potential of the cells.

### Live-Cell Microscopy

CDC20-deficient RPE-1 cells expressing H2B-GFP to allow the visualization of the cell cycle were sorted based on GFP fluorescence and then plated in collagen-coated 96-well microplates with black well walls and an optically clear cyclic olefin bottom for high-content analysis. Approximately 1,200–1,500 cells were plated in each well. Plates were mounted on an inverted microscope equipped with the PerkinElmer Operetta high-content imaging system. The microscope was enclosed within a temperature- and CO_2_- controlled environment that maintained an atmosphere of 37°C and a humidified 5% CO_2_ atmosphere. Wells containing H2B-GFP-expressing cells of interest were identified manually, and fluorescence and differential interference contrast images were captured every 20 min using the selected fluorescence objective for up to 72 h. All captured images were analyzed using Harmony software to track the progression of the cell cycle, calculate the cell number, and so forth.

### The Long-Term Culture of the Tumorigenesis Model

Animal experiments were approved by the Shanghai Jiao Tong University Animal Care and Use Committee and were conducted following the animal policies of Shanghai Jiao Tong University in accordance with the guidelines established by the National Health and Family Planning Commission of China. Normal cells were first transfected with the CDC20 lentiviral vector and then transfected with the H2B-EGFP plasmid containing the CytoMegalo virus (CMV) promoter, and the mitosis process was visualized. Glucose- and glutamine-free DMEM (GIBCO) was supplemented with 5% FBS, 1% penicillin/streptomycin, and glutamine (configured to three concentration gradients of 2 mM, 4 mM, and 6 mM) and mixed at 37°C, and the microenvironment was adjusted to three pH values (pH 7.0, pH 6.6, and pH 6.2). The newly constructed stable transgenic cells were plated at a density of 1 × 10^6^ in a 6-cm dish, cultured overnight, and directly transferred to culture medium mimicking a tumor-like microenvironment. When the cells reached a density of 90%, they were trypsinized and passaged. When the cells had regrown, we trypsinized the cells to a density of 1 × 10^6^ cells per milliliter. Each mouse was subcutaneously injected with 100 μL of the cells. One flank of each mouse was injected with the tumor cells as the experimental group, and the other flank was injected with the vehicle as a control group. We observed the formation of subcutaneous tumors at the injection site in the mice weekly and recorded the initial time of tumor formation and the weekly growth thereafter.

### Analyses of RNA-Seq Data and Self-Organizing Maps

Cells were collected at different stages, and RNA-seq was performed. Then, we used the first 4,000 genes in the library to train the self-organizing map. Prior to SOM training, the data were normalized on a gene-by-gene basis by subtracting the average of each vector and dividing by its standard deviation. The map grid was initialized and multiplied by the first two main components of the data using a sine function to produce a smooth circular boundary condition. The training lasted for 200 cycles, during which the unit adapted to the radius of the winning unit, where h represents the height of the map (the direction in which the maximum length is always selected) to define the python code for further analysis, including clustering and visualization. The cluster was identified by the local minima of the u matrix, and the value of each cell was defined as the average of the vector differences between the cell prototype and the six neighbors on the si-square grid. All other unit prototypes were then assigned to the cluster based on the minimum vector distance to the seed unit.

A GSEA was performed, and differently expressed genes were analyzed.

### Statistical Analysis

All experiments were performed in triplicate. All statistical analyses were conducted using SPSS v.19.0 software. The statistical analysis was performed with a double-sided Student’s t test for comparisons of two groups. All data are presented as mean ± standard error of the mean (SEM). Differences at p < 0.05 were considered statistically significant.

## Author Contributions

Q.G. and F.L. conducted the experiments and wrote the paper. F.Z. analyzed the data and revised the paper. S.G., R.J., and X.F designed the experiment and instructed the study. All authors read and approved the final manuscript.

## Conflicts of Interest

The authors declare no competing interests.

## Supplemental Information

Document S1. Figure S1

Document S2. Article plus Supplemental Information

## References

[1] Cairns R.A., Harris I.S., Mak T.W. (2011). Regulation of cancer cell metabolism. Nat. Rev. Cancer.

[2] Chiche J., Brahimi-Horn M.C., Pouysségur J. (2010). Tumour hypoxia induces a metabolic shift causing acidosis: a common feature in cancer. J. Cell. Mol. Med..

[3] Warburg O. (1956). On the origin of cancer cells. Science.

[4] Gillies R.J., Robey I., Gatenby R.A. (2008). Causes and consequences of increased glucose metabolism of cancers. J. Nucl. Med..

[5] Matoba S., Kang J.G., Patino W.D., Wragg A., Boehm M., Gavrilova O., Hurley P.J., Bunz F., Hwang P.M. (2006). p53 regulates mitochondrial respiration. Science.

[6] Nasmyth K., Haering C.H. (2009). Cohesin: its roles and mechanisms. Annu. Rev. Genet..

[7] Musacchio A., Salmon E.D. (2007). The spindle-assembly checkpoint in space and time. Nat. Rev. Mol. Cell Biol..

[8] Burrell R.A., McClelland S.E., Endesfelder D., Groth P., Weller M.C., Shaikh N., Domingo E., Kanu N., Dewhurst S.M., Gronroos E. (2013). Replication stress links structural and numerical cancer chromosomal instability. Nature.

[9] Crasta K., Ganem N.J., Dagher R., Lantermann A.B., Ivanova E.V., Pan Y., Nezi L., Protopopov A., Chowdhury D., Pellman D. (2012). DNA breaks and chromosome pulverization from errors in mitosis. Nature.

[10] Musacchio A. (2015). The molecular biology of spindle assembly checkpoint signaling dynamics. Curr. Biol..

[11] Lara-Gonzalez P., Westhorpe F.G., Taylor S.S. (2012). The spindle assembly checkpoint. Curr. Biol..

[12] Gordon D.J., Resio B., Pellman D. (2012). Causes and consequences of aneuploidy in cancer. Nat. Rev. Genet..

[13] Reichert M., Steinbach J.P., Supra P., Weller M. (2002). Modulation of growth and radiochemosensitivity of human malignant glioma cells by acidosis. Cancer.

[14] Mathew R., Karp C.M., Beaudoin B., Vuong N., Chen G., Chen H.Y., Bray K., Reddy A., Bhanot G., Gelinas C. (2009). Autophagy suppresses tumorigenesis through elimination of p62. Cell.

[15] Mathew R., Kongara S., Beaudoin B., Karp C.M., Bray K., Degenhardt K., Chen G., Jin S., White E. (2007). Autophagy suppresses tumor progression by limiting chromosomal instability. Genes Dev..

[16] Degenhardt K., Mathew R., Beaudoin B., Bray K., Anderson D., Chen G., Mukherjee C., Shi Y., Gélinas C., Fan Y. (2006). Autophagy promotes tumor cell survival and restricts necrosis, inflammation, and tumorigenesis. Cancer Cell.

[17] Kastan M.B., Bartek J. (2004). Cell-cycle checkpoints and cancer. Nature.

[18] Meek D.W., Anderson C.W. (2009). Posttranslational modification of p53: cooperative integrators of function. Cold Spring Harb. Perspect. Biol..

[19] Shieh S.Y., Ikeda M., Taya Y., Prives C. (1997). DNA damage-induced phosphorylation of p53 alleviates inhibition by MDM2. Cell.

[20] Kubbutat M.H., Jones S.N., Vousden K.H. (1997). Regulation of p53 stability by Mdm2. Nature.

[21] Hanahan D., Weinberg R.A. (2011). Hallmarks of cancer: the next generation. Cell.

[22] Nakazawa M.S., Keith B., Simon M.C. (2016). Oxygen availability and metabolic adaptations. Nat. Rev. Cancer.

[23] Sennino B., McDonald D.M. (2012). Controlling escape from angiogenesis inhibitors. Nat. Rev. Cancer.

[24] Bertout J.A., Patel S.A., Simon M.C. (2008). The impact of O_2_ availability on human cancer. Nat. Rev. Cancer.

[25] Peppicelli S., Bianchini F., Calorini L. (2014). Extracellular acidity, a “reappreciated” trait of tumor environment driving malignancy: perspectives in diagnosis and therapy. Cancer Metastasis Rev..

[26] Robertson-Tessi M., Gillies R.J., Gatenby R.A., Anderson A.R. (2015). Impact of metabolic heterogeneity on tumor growth, invasion, and treatment outcomes. Cancer Res..

[27] Hamanaka R.B., Chandel N.S. (2010). Mitochondrial reactive oxygen species regulate cellular signaling and dictate biological outcomes. Trends Biochem. Sci..

[28] Orvedahl A., Levine B. (2009). Eating the enemy within: autophagy in infectious diseases. Cell Death Differ..

[29] Meijer A.J., Codogno P. (2009). Autophagy: regulation and role in disease. Crit. Rev. Clin. Lab. Sci..

[30] Gao P., Sun L., He X., Cao Y., Zhang H. (2012). MicroRNAs and the Warburg effect: new players in an old arena. Curr. Gene Ther..

