# Supplementary material for: CDC20 Knockdown and Acidic Microenvironment Collaboratively Promote Tumorigenesis through Inhibiting Autophagy and Apoptosis
Source: Mol Ther Oncolytics. 2020 Mar 30;17:94–106. doi: 10.1016/j.omto.2020.03.015 (PMC7163048; doi:10.1016/j.omto.2020.03.015)

OMTO, Volume *17*

Supplemental Information

CDC20 Knockdown and Acidic Microenvironment Collaboratively Promote Tumorigenesis through Inhibiting Autophagy and Apoptosis

Qingying Gu, Fang Li, Shengfang Ge, Feifei Zhang, Renbing Jia, and Xianqun Fan

Supplement

Figure S: (A&B) The efficiency of CDC20 knockdown of sh3 in three cell lines and the statistical result was shown. (C&D)a gradient acidity experiment to screen for the most suitable one for long-term domestication experiments. (E) LC3, p62, ATG5, p-S6K and S6K expression levels of Mock and shCDC20 FHC cells in different medium were detected by western blot. (F) LC3, p62, ATG5, p-S6K and S6K expression levels of Mock and shCDC20 FHC cells in different medium were detected by western blot. (G) Pro-caspase 3, cleaved-caspase 3 and p53 of 2M, 2C, 3M, 3C, 4M and 4C FHC cells were detected using western blot. (H) Pro-caspase 3, cleaved-caspase 3 and p53 of 2M, 2C, 3M, 3C, 4M and 4C FHC cells were detected using western blot.

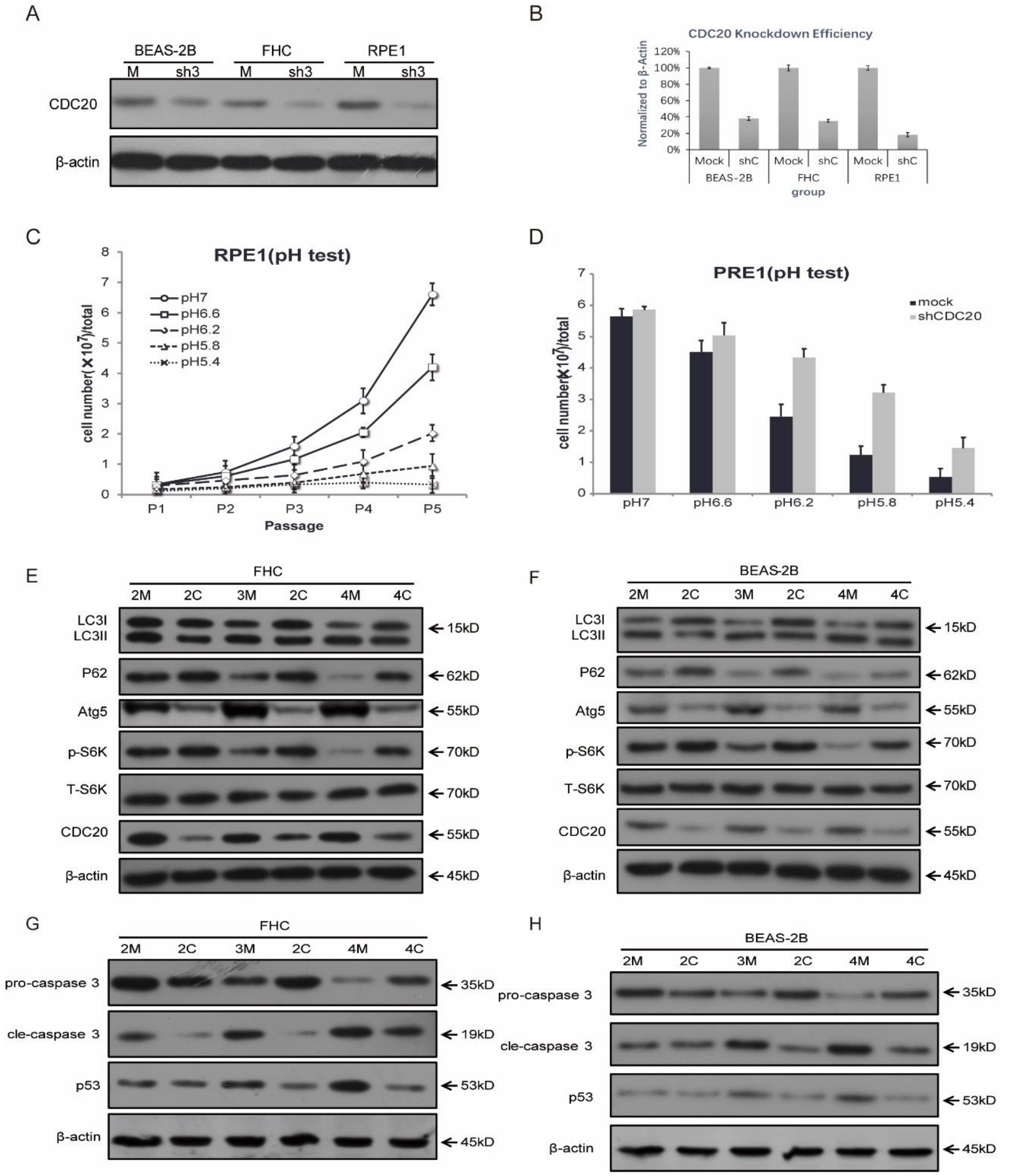

Supplement: Document S1. Figure S1 [file mmc1.pdf]
